# The different manifestations of ^18^F-FDG PET/CT and ^68^Ga-FAPI-04 PET/CT in evaluation of the steroid therapy response for IgG4-related disease: A case report

**DOI:** 10.3389/fnume.2022.1038797

**Published:** 2023-01-04

**Authors:** Guorong Jia, Dejian Bian, Chao Cheng, Meitang Wang, Changjing Zuo

**Affiliations:** ^1^ Department of Nuclear Medicine, Shanghai Changhai Hospital, Navy Military Medical University, Shanghai, China; ^2^ Department of Emergency, Shanghai Changhai Hospital, Navy Military Medical University, Shanghai, China

**Keywords:** ^18^F-FDG, ^68^Ga-FAPI-04, IgG4-related disease, PET/CT, therapy

## Abstract

IgG4-related disease is a fibrous-inflammatory process belonging to immunomodulation disorders. We report a case of a 57-year-old man with the IgG4-related disease (RD). ^68^Ga-FAPI-04 PET/CT showed more significant uptake in most lesions than in ^18^F-FDG PET/CT except for the cervical and mediastinal lymph nodes. Besides, uptake in the submandibular glands were only detected in ^68^Ga-FAPI-04 PET/CT. The biopsy result of the cervical lymph nodes confirmed the diagnosis of IgG4-related disease. After treatment, only slight FDG-avid cervical lymph nodes were observed in the ^18^F-FDG PET/CT, while the raised uptake of ^68^Ga-FAPI-04 could be observed in the pancreas and submandibular glands. ^68^Ga-FAPI-04 PET-CT might have promising applications in evaluating IgG4-RD, whether in initial or follow-up imaging during steroid therapy.

## Introduction

IgG4-related disease (RD) is an autoimmune-mediated disorder that involves different organs. A large amount of immune cell infiltration of the IgG4-related disease induced a focal mass that mimics a malignancy tumor in imaging examination (1). Especially in the pancreas, autoimmune pancreatitis has sometimes been misdiagnosed as pancreatic cancer (2). It is of great importance to distinguish IgG4-RD from tumors. Previous studies have validated the utility of ^18^F-fluorodeoxyglucose (^18^F-FDG) PET/CT in the differential diagnosis (3). Fibroblast activation protein is not only present in tumor stroma but also in some benign lesions with prominent fibroblast proliferation. ^68^Ga-labeled fibroblast activation protein inhibitor (^68^Ga-FAPI) is a novel PET agent. Some researchers have highlighted that ^68^Ga-FAPI PET/CT would contribute to diagnosing IgG4-related disease (RD) (4), but few studies to date have compared the role of ^18^F-FDG PET/CT and ^68^Ga-FAPI PET/CT in the follow-up of IgG4-RD. Here, we reported a case comparing the pre- vs. post- and ^18^F-FDG vs. ^68^Ga-FAPI-04 images of an IgG4-RD patient who underwent the prednisone treatment.

## Case description

**Figure 1:**
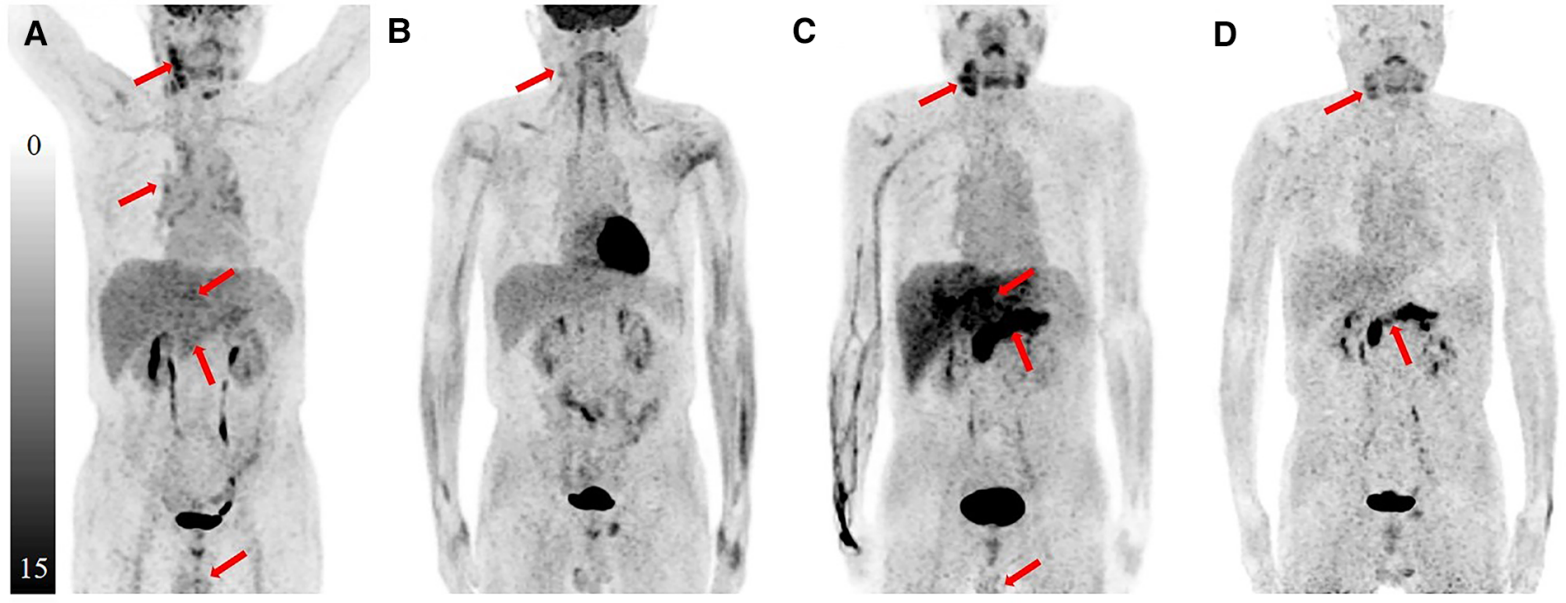
The MIP images of ^18^F-FDG PET/CT and ^68^Ga-FAPI PET/CT. The pre-treatment ^18^F-FDG PET/CT (**A**) showed elevated uptake of the following lesions: cervical and mediastinal lymph nodes, intrahepatic bile ducts, pancreas, and prostate. The pre-treatment ^68^Ga-FAPI PET/CT (**C**) indicated more obvious uptake in most lesions mentioned above except for the cervical and mediastinal lymph nodes; besides that, submandibular glands were also involved. After prednisone treatment, FDG-avid lesion was detected only in the right cervical lymph node (**B**), but the raised uptake of ^68^Ga-FAPI could still be observed in the pancreas and submandibular glands (**D**).

**Figure 2:**
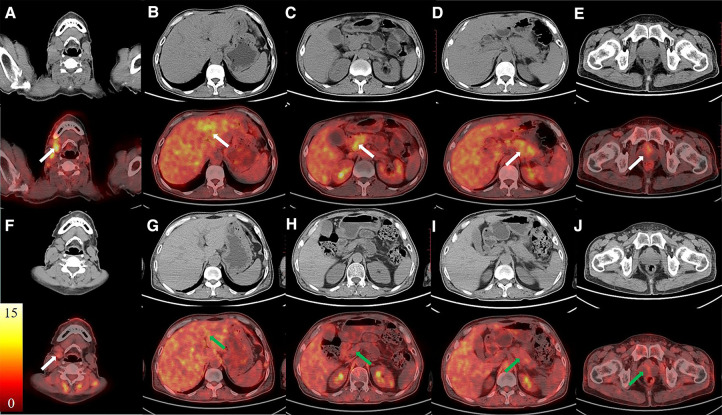
The images of ^18^F-FDG PET/CT. The CT images were in the first/third rows, and the fused PET/CT images were in the second/fourth rows. The pre-treatment images (**A–E**, white arrows) showed elevated uptake of the following lesions: cervical lymph nodes, intrahepatic bile ducts, pancreas, and prostate. FDG-avid lesion was detected only in the right cervical lymph node after the treatment (**F–J**).

**Figure 3:**
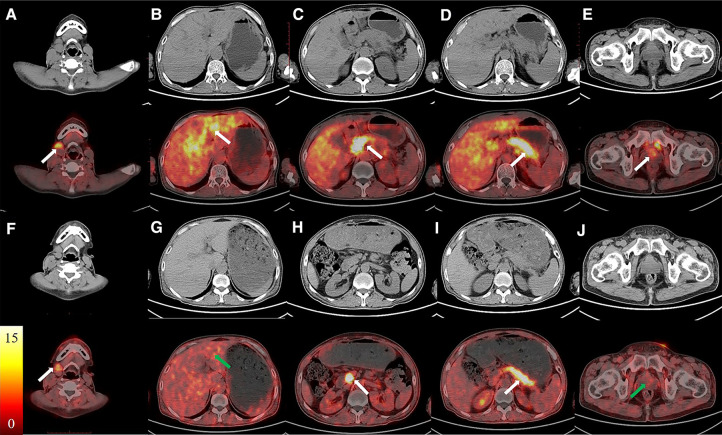
The images of ^68^Ga-FAPI PET/CT. The CT images were in the first/third rows, the fused PET/CT images were in second/fourth rows. The pre-treatment images (**A–E**, white arrows) showed elevated uptake of submandibular glands, intrahepatic bile ducts, pancreas, and prostate. The raised uptake of ^68^Ga-FAPI could still be observed in the submandibular glands and pancreas after treatment (**F–J**, white arrows).

A 57-year-old man with jaundice for 4 months, abdominal pain and diarrhea for 2 months was presented to the emergency department. He was diagnosed with suspected cholangiocarcinoma because of the thickening of the bile duct wall observed on contrast-enhanced CT. The ^18^F-FDG PET/CT and ^68^Ga-FAPI-04 PET/CT were executed for the differential diagnosis and possible tumor staging (Figure 1 Maximum intensity projection images of ^18^F-FDG and ^68^Ga-FAPI-04 PET/CT). The pre-treatment ^18^F-FDG PET/CT pictures (Figures 2A–E) showed elevated uptake of the following lesions: cervical (SUVmax 4.7) and mediastinal lymph nodes (SUVmax 2.7), intrahepatic bile ducts (SUVmax 3.3), pancreas (SUVmax 3.0), and prostate (SUVmax 2.3). ^68^Ga-FAPI-04 PET/CT was also performed, and higher uptake in most lesions mentioned above was observed except for the cervical and mediastinal lymph nodes, Besides that, submandibular glands were also involved (Figures 3A–E). The patient's total bilirubin was elevated to 94.7 µmol/L. The tumor marker CA19-9 was 204.63 U/ml. The amylase was 228 U/L, and the IgG4 was 26.2 g/L (reference range: 0.03–2.1 g/L). A loco-regional lymphadenectomy and biopsy were performed. According to the biopsy result of the cervical lymph nodes, the final diagnosis was classified as an IgG4-RD. Subsequently, treatment with oral prednisone was initiated. The initial dose was 40 mg/d which lasted for 2 weeks. Then reduced by 5 mg every week, until the final dose of 5 mg/d which was used for 6 months. One month after treatment with prednisolone, the value of IgG4 was decreased to 13.6 g/L, CA19-9 to 36.94 U/ml, and the total bilirubin was reduced to 29.8 µmol/L. The symptoms of jaundice were relieved remarkably. During the same period of time, a slight FDG-avid lesion was detected only in the right cervical lymph node (SUVmax 1.7) (Figures 2F–J), but the raised uptake of ^68^Ga-FAPI-04 could still be observed in the pancreas (SUVmax 5.6) and submandibular glands (SUVmax 2.3) (Figures 3F–J).

## Discussion

The FDG accumulation is the reflection of glucose metabolism in the tumor or inflammation lesion. Considerable evidence has demonstrated the ability of ^18^F-FDG PET to assess IgG4-RD both at initial evaluation and after therapy (2, 3). ^68^Ga-FAPI is thought to be the potential broad-spectrum tumor PET agent targeting FAP (5). Fibroblasts were activated in some benign diseases (6), such as IgG4-RD, and it can also be FAPI-positive.

In IgG4-RD, large quantities of fibroblasts lead to fibrosis in the extracellular matrix (7, 8). Some case reports and articles have also explored the utility of ^68^Ga-FAPI PET/CT to assess IgG4-RD (8, 9). One study of 26 IgG4-RD patients revealed that ^68^Ga-FAPI PET/CT detected more involved organs in 13 (50.0%) patients and significantly higher SUV than that of ^18^F-FDG PET/CT (4). However, FDG-avid lymph node did not accumulate ^68^Ga-FAPI (4, 10). The two results were both validated in this case report. The mismatch of ^68^Ga-FAPI and ^18^F-FDG revealed that the lesion was probably in different stages; the FDG-avid lesion was inflammatory-proliferative, while the ^68^Ga-FAPI positive lesion was likely in a fibrotic phase. Another possible pathological reason is that fibrosis is rare in most lymphadenopathy patterns of IgG4-RD (11). Our previous study about pancreatic cancer found that ^68^Ga-FAPI-04 PET/CT detected more positive lymph nodes whose activity was over background than ^18^F-FDG PET/CT (12). The critical parameters of ^18^F-FDG PET/CT for diagnosing malignant metastatic lymph nodes have been highly explored such as the SUVmax cut-off point, groups of lymph nodes, and the SUV value of tumor (13). But the utility of ^68^Ga-FAPI in detecting malignant metastatic lymph nodes is still in the stage of development and in comparison with ^18^F-FDG (14).

After the prednisone treatment, slight uptake in cervical lymph node was only observed in ^18^F-FDG PET/CT, whereas uptake in pancreas and submandibular glands were more noticeable in the ^68^Ga-FAPI-04 PET/CT. The laboratory test result of IgG4 of 13.6 g/L, which was also above the reference range, partly confirmed the involvement of IgG4-RD. In another cross-sectional clinical study with inflammatory, fibrotic and overlapping manifestations of IgG4-related disease, the responsiveness to immunosuppressive therapy was more sensitive in inflammatory lesions than in fibrotic lesions (15). This reason might explain the difference in ^68^Ga-FAPI-04 and ^18^F-FDG imaging after treatment with prednisone.

Notwithstanding its limitation, our case report found that ^68^Ga-FAPI-04 could provide additional insights into IgG4-RD beyond the inflammation process that demonstrated by ^18^F-FDG. The combination of ^18^F-FDG and ^68^Ga-FAPI-04 may demonstrate even greater potency in the efficacy assessment of IgG4-RD in the future.

## Data Availability

The original contributions presented in the study are included in the article/Supplementary Material, further inquiries can be directed to the corresponding author/s.
